# Construction of English and American Literature Corpus Based on Machine Learning Algorithm

**DOI:** 10.1155/2022/9773452

**Published:** 2022-06-02

**Authors:** Qian Dai

**Affiliations:** School of Foreign Languages, Henan Polytechnic University, Jiaozuo 454003, Henan Province, China

## Abstract

In China, the application of corpus in language teaching, especially in English and American literature teaching, is still in the preliminary research stage, and there are various shortcomings, which have not been paid due attention by front-line educators. Constructing English and American literature corpus according to certain principles can effectively promote English and American literature teaching. The research of this paper is devoted to how to automatically build a corpus of English and American literature. In the process of keyword extraction, key phrases and keywords are effectively combined. The similarity between atomic events is calculated by the TextRank algorithm, and then the first *N* sentences with high similarity are selected and sorted. Based on ML (machine learning) text classification method, a combined classifier based on SVM (support vector machine) and NB (Naive Bayes) is proposed. The experimental results show that, from the point of view of accuracy and recall, the classification effect of the combined algorithm proposed in this paper is the best among the three methods. The best classification results of accuracy, recall, and *F* value are 0.87, 0.9, and 0.89, respectively. Experimental results show that this method can quickly, accurately, and persistently obtain high-quality bilingual mixed web pages.

## 1. Introduction

In recent years, English and American literature has been paid more and more attention as a professional course to improve the humanistic quality and basic language skills of English majors. English and American literature course plays a very important role in the training plan of English majors in colleges and universities in China. Reading and analyzing a certain number of British and American literary works can improve students' basic language skills and humanistic quality and enhance their understanding of western literature and culture. As the course of English and American literature involves historical and cultural background, literary history, appreciation of original works, and literary theory and its contents are complex, it is inevitable that there will be a phenomenon of “cramming, ordering, and passing by” in the teaching process, and learners lack interest and initiative in learning. To understand British and American culture, it is more important to improve and cultivate students' autonomous learning ability, cultivate their literary appreciation and aesthetic sensitivity, and promote the cultivation of humanistic quality as a whole.

With the wide application of statistical methods, large-scale corpus has become an indispensable basic resource in the field of natural language processing. Natural language processing technologies developed based on statistical methods include hidden Markov model, maximum entropy model, Bayesian method, SVM (support vector machine), and other mining methods, which can be compared with this paper [1–3]. Based on this assumption, pages in different languages on the network have similar document structures, such as titles and paragraphs, if their contents are comparable. Stasak et al. proposed a comparable text mining method based on statistical information on word frequency distribution, which is independent of the construction language [4]. Meng et al. translated the words in the source language pages through a bilingual dictionary, then used these words to construct a query, and retrieved the first *N* (fixed value) related documents from the acquired target language page set [5]. Hassanpour and Langlotz crawled the monolingual culture through the designated website and adopted a method similar to Talvensaari to obtain a comparable corpus [6]. Sun and Du adopted the improved minimum editing distance algorithm and achieved good results in the experiment [7]. The above methods, with the help of machine translation and cross-language retrieval technology, have achieved certain results in obtaining comparable pages. However, at present, there are few researches on English and American literature corpus retrieval technology, and there are no referential results.

Corpus usually refers to the language materials collected for language research and stored in electronic form. It is a basic resource that is collected from naturally occurring written or spoken language samples, scientifically selected and marked, has an appropriate scale, and can reflect and record the actual use of language. Many corpora have been established in China in recent years, but most of them are used for linguistic research, grammar research, dictionary compilation, textbook compilation, or research in a specific field [8]. The corpus of three sources of English and American literary and cultural context is quite different from other existing corpora. In the past, the materials in the corpus were collected and sorted out manually. Today, due to the use of advanced computer technology, the efficiency and scale of corpus construction have been greatly improved, laying a solid foundation for corpus construction and wider application. At present, the construction of English and American literature corpus in China mainly focuses on the research of alignment technology and application technology. In recent years, the systematic construction of English and American literature corpus has gradually increased. The significance of this paper lies in learning the technical achievements of predecessors and exploring the construction process of English and American literature corpus through actual programming so as to apply what we have learned.

## 2. Related Work

### 2.1. Overview of ML Theory

In recent years, ML (machine learning) algorithm has received unprecedented attention and has been widely used in personalized recommendation, speech recognition, spam filtering, face detection, protein structure prediction, vehicle control, and medical diagnosis.

Liu et al. used the data parallel strategy to split a large data set into several small data sets for parallel local clustering and synchronously generated a global clustering center after the local clustering was finished [9]. The experimental results show that the parallel clustering algorithm has a short time cost in the process of large-scale data clustering. Huang et al. found through theoretical analysis of the modified classical ML algorithm that the new algorithm is not an approximate solution of the original algorithm, but an exact solution, and the distributed implementation technology can have linear scalability with the increase of cluster scale [10]. Babu and Suresh proposed a parallel logistic regression algorithm based on the Spark platform [11]. In this study, the data sets that are reused in logistic regression are cached in memory by the Spark platform, and the parameters are optimized by distributed computing gradient descent. Ajitha et al. distributed data and linear algebra operations in a number of cluster nodes for parallel execution, and at the same time, the main operations were mapped into the parallel computation of matrix multiplication based on the principle of data locality by using multithread architecture in cluster nodes [12]. The experimental results show that the implementation of parallel algorithm based on hybrid architecture is faster than the simple implementation.

Zhong et al. proposed an online learning algorithm for SVM, which is used to deal with the classification problem of gradually providing input data in sequence [13]. The algorithm is faster, uses fewer support vectors, and has better generalization ability. Yuvaraj et al. put forward a fast, numerically stable, and robust incremental SVM learning method [14]. Abualhaija et al. put forward a feature selection method for classification based on SVM. The accuracy of the SVM classification algorithm is related to the number of features and the size of data sets, so selecting features of data before classification is beneficial to improve the classification accuracy [15]. However, the feature selection method is also very important, and the features selected by different feature selection methods are very different. Tsai and Chang put forward an algorithm to build a decision tree classifier. This algorithm runs in a distributed environment and is suitable for large data sets and streaming data. Compared with serial decision trees, this method can improve the efficiency on the premise of approximate accuracy error [16].

### 2.2. Research Corpus

The application fields of natural processing mainly include automatic question answering system, machine translation, speech recognition, document summarization, and document classification. With the popularity of electronic devices and the growth of the World Wide Web, the corpus has been expanding in scale. At first, the corpus had little content, and the capacity of words was very small, which basically did not reach more than one million words, and it was all based on the research of English in various countries' linguistics. Researchers found and constructed random models from the speech corpus, which made it possible to realize the speech recognition function.

Dattner extracts the key information in the source language document and then queries it through the information retrieval system after translation. In order to improve the alignment effect, the retrieval results are filtered [17]. Knight constructs a corresponding URL(Uniform resource locator), and if there is such a URL, it is used as a candidate web page pair. After manual evaluation of the web pages obtained by this system, the accuracy rate is nearly 90%, and the obtained English texts are 137 M and 117 M [18]. Mori et al. marked the reference information and text type of the text, divided the text chapters, paragraphs, and sentence boundaries, automatically aligned the sentences in the original text and the translated text, and then corrected them manually [19]. Charles made a more in-depth study on the structural similarity of parallel web pages, adding more features to eliminate the nonparallel web page pairs among the candidate parallel web pages. When manually evaluating about 400 pairs of Chinese-English parallel web pages, about 3,500 pairs of Chinese-English parallel web pages were obtained, with an accuracy rate of 98% and a recall rate of 61% [20].

Mereu carried out various types of searches, including searches based on words, word frequencies, and sentence patterns. The Chinese-Japanese subdatabases in this English-American literature corpus are aligned at the segment level [21]. Ozn people put forward a feature that can automatically discover the parallel web page pairs named by the authors of the current site and then obtain the candidate parallel web page pairs. This method does not need to define language-related string sets in advance [22].

## 3. Research Method

### 3.1. Corpus Extraction

The corpus of English and American literature courses is designed to provide sufficient information sources for English and American literature teachers and learners so as to help improve the teaching effect of English and American literature courses and learners' interest in learning and enhance learners' comprehensive quality and appreciation ability. The principle of corpus construction should be based on meeting the teaching objectives of British and American courses. Therefore, when constructing the corpus, according to the needs of this part of learners, the knowledge can be concentrated and systematized, and all relevant contents not covered in the class can be included in the corpus to meet the needs of learners. According to this theory, we decided to adhere to the principle of student-centered and teacher-led and highlight the elements of environmental design, meaning construction, and interactive learning to build a literary corpus.

The construction of literary corpus should highlight literariness, embody teaching content, emphasize the literary knowledge of English and American literature from background to original works, and avoid taking the public as the object and taking the examination as the guide. Literary corpus needs to be prominent, systematic, centralized, and interactive. For example, for students majoring in English language and literature, they should not only master the knowledge of English and American literature in an all-round way, but also know the background knowledge of English and American culture in literary works. This kind of corpus-based literature teaching can not only provide rich and authentic language patterns for language and literature learners but also make it easier for teachers to guide students to conduct in-depth study and further research because of the multidimensional, integrated, and interactive nature of computers and the powerful functions of software.

In this paper, keywords are extracted from source language documents and translated and used for information retrieval to retrieve target language documents related to translated keywords. This process is equivalent to indirectly using the extracted keywords for information retrieval. Automatic keyword extraction provides convenience for quick browsing and application of these resources. The existing keyword extraction methods can be roughly divided into two types: supervised and unsupervised.

**Figure 1:**
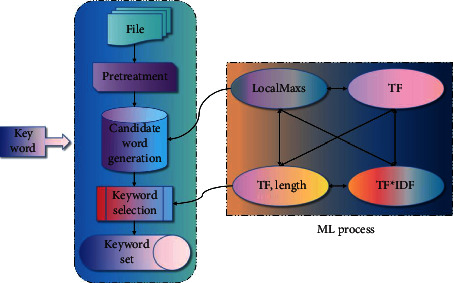
Keyword extraction process.

This paper presents a keyword extraction method based on multiword expression and related word ranking. In order to make the extracted keywords more suitable for retrieval, this method pays more attention to the construction of candidate phrases and combines key phrases with keywords.  Figure 1 describes the overall process of keyword extraction in this paper.

It mainly includes three modules: preprocessing, candidate set construction, and keyword selection. Because of the characteristics of English texts, it is necessary to analyze the source language documents first, then construct the candidate sets of phrases and single words respectively, sort the candidate words, and finally select the candidate words with the highest weight as the keyword set.

Text diagram is a formal model to describe the relationship between texts. It is a diagram structure in which some feature items in a text are vertices, and the relationship between features is edges. The graph-based sorting algorithm applied in the field of natural language processing is called TextRank.

The general TextRank model can be expressed as a weighted directed graph *G*=(*V*, *E*), which consists of a set of points *V* and an edge set *E*. The weight of the edge between two vertices (*i*, *j*) in the graph is noted as *w*_*ij*_.

For a given vertex *V*_*i*_, In(*V*_*i*_) is expressed as the set of points pointing to that point, and Out(*V*_*i*_) is the set of points that the *V*_*i*_ point points to other points, as shown in the following formula (1):

(1) $$\begin{array}{c} WS\left(V_{i}\right)=\left(1-d\right)+d*\sum_{V_{j}\in In\left(V_{i}\right)}\frac{w_{ji}}{\sum_{V_{k}\in \text{Out}\left(V_{j}\right)}w_{jk}}WS\left(V_{j}\right). \end{array}$$

*w*_*ji*_ represents the similarity of two sentences, *WS*(*V*_*j*_) represents the weight of the last iteration *J*, and the whole formula is an iterative process.

For bilingual parallel resource pairs, a character in language *S*_1_ is related to a random number of characters in another language *S*_2_, and these random numbers are not only independent of each other but also in accord with normal distribution. The model of normal distribution is determined by the median *c* and variance *s*^2^. Define the following formula to calculate the length-based measure *δ* to meet the expected value of 0 and the normal distribution with variance of 1.

(2) $$\begin{array}{c} \delta =\frac{l_{2}-l_{1}c}{\sqrt{l_{1}s^{2}}}, \end{array}$$

where *l*_1_, *l*_2_ denote the length of sentences *S*_1_, *S*_2_ (i.e., the total number of words).

From the above, it can be seen that the construction process of candidate phrases and candidate single words is independent of each other, and the phrases and single words have different characteristics, so the correlation ranking process is also carried out separately.

### 3.2. Automatic Acquisition of Parallel Resources in English and American Literary Corpora

In the field of machine translation, bilingual parallel corpus has been widely concerned by researchers in recent years. British-American literary corpus can also be used to improve learning efficiency in bilingual teaching, and linguists can study the development trend of language and characters. Therefore, the corpus of English and American literature is a very good research direction. Particularly in bilingual mixed web pages, parallel resources always appear in similar or even identical layout. Based on this phenomenon, this section proposes a completely adaptive method to mine parallel resources.

In order to obtain parallel resource pairs from the web data domain, we should first cut the text into continuous segments according to the language and other pieces of information, then mine a few typical parallel text segment pairs based on the length model, alignment model, and symbol features, and use them as seeds for the next task. Once the wrapper is built, more potential parallel resources can be obtained by positive expression matching (i.e., the parallel resources are abandoned because they do not conform to the length model or the alignment model when the seed mining task is performed).

When constructing the multiview semantic tree database, an important goal is to keep the compatibility between the grammar tree and the semantic tree, which involves the transmission of empty components. After the transmission, the transmitted components semantically act as predicates that have no direct jurisdiction with them; in other words, they involve either verbs parallel to the current verbs or verbs that govern the current verbs. We name these two kinds of transmission as semantic role transmission within verbs and semantic role transmission between verbs, and they can be used in combination.

**Figure 2:**
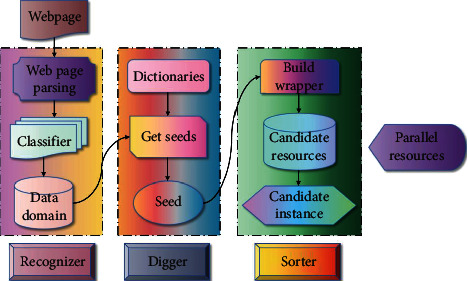
System flow of automatic construction of English and American literature corpus on mixed web pages.

The method in this chapter can be applied to mixed web pages described by any language pair; that is, it can be used to obtain parallel resources between any language pairs. The fundamental reason is that this method does not need any expert knowledge related to language, domain, and so on.  Figure 2 shows the system flow of automatic construction of English and American literature corpus.

In the abstract of the page, many translations of unknown words are embedded in an English sentence; that is, they contain interfering words. If all the components of an unknown word can be translated by a bilingual dictionary, the translation combination query will be conducted. The cooccurrence model makes use of the number of cooccurrence words of unknown words and their candidate translations in page abstracts, and the specific definition is shown in formula (3):

(3) $$\begin{array}{c} \text{Fre}\left(E_{i}^{*}\right)=\frac{f\left(E_{i}^{*}\right)}{\underset{j\in \left[1,n\right]}{\max\left(f\left(E_{i}^{*}\right)\right)+1}}, \end{array}$$

where *f*(*E*_*i*_^*∗*^) is the cooccurrence times of candidate translations and unlisted words and *n* is the number of candidate translations.

Map document *D* through vector space to get the weight information of word items, and the expression form of document *D* is converted into *D*={*T*_1_, *W*_1_; *T*_1_, *W*_1_; …; *T*_*n*_, *W*_*n*_}, where *W*_*n*_ represents the weight of each word item. At this point, we can get the vector expression form of document *D*, abbreviated as *D*=*D*(*W*_1_, *W*_2_,…, *W*_*n*_).

In the vector space model, the content correlation degree *S*(*D*_1_, *D*_2_) between two documents *D*_1_, *D*_2_ is usually expressed by the cosine of the angle between vectors, and the formula (4) is

(4) $$\begin{array}{c} S\left(D_{1},D_{2}\right)=\cos\;\theta =\frac{\sum_{i=1}^{n}W_{1i}\times W_{2i}}{\sqrt{\left(\sum_{i=1}^{n}W_{1i}^{2}\times W_{2i}^{2}\right)}}. \end{array}$$

As all events have obvious event elements such as time, place, and people, most of the features are named entities. These feature items are converted into vector form, and the content similarity is calculated by formula (4). Finally, the similarity of texts is measured by summing the time similarity and the content similarity.

### 3.3. Text Classification

There are many kinds of textbooks for English and American literature courses. In order to provide learners with more reference range, when constructing corpus, we should expand the scope of material selection, make use of the superior resources of the Internet at present, and establish hyperlinks of literary background and literary criticism. In addition to using various resources to collect the necessary cultural background and literary knowledge materials for English and American literature teaching, we should also set up tracking corpus to form a complete corpus system so as to help teach workers to grasp the characteristics and overall picture of language learners' learning as a whole.

Text classification is to determine the classification of all unknown documents according to the predetermined topic categories so as to realize the objective processing of texts and achieve the goal of improving the accuracy of classification. As a tool for processing information, it perfectly presents efficient classification algorithms and accurate query results in the field of information retrieval. Text classification can generally be divided into single label and multilabel. The single label means that an article belongs to only one category, while multilabel means that an article may belong to both one category and multiple categories at the same time. This topic only involves the task of single label text classification.

Literary corpus can be used to study literature, especially the style of writers. The unique computer retrieval and statistical technology can accurately count the word frequency, word length, and sentence length of a writer's works, which can reflect the writer's writing style and literary background in a certain period. ML refers to the method of feature extraction based on the idea of word frequency, and classification algorithms such as SVM, *K*-nearest neighbor method, and NB (Naive Bayes) method are used to classify texts. Every word in English is connected by spaces, so its word segmentation can be completed by using spaces.

From the perspective of system workflow, the system workflow in the prototype of text classification demonstration is as follows:

1. The training text is segmented, and stop words are removed to obtain the initial text feature information.
2. Complete the text representation operation to obtain the text feature vector for the training of the classifier.
3. Classify the text; that is, first classify the text with SVM classifier, and then classify the text with NB classifier for the second time so as to get the classification result.
4. Evaluate the final result of system classification and determine the classification effect of system prototype.

**Figure 3:**
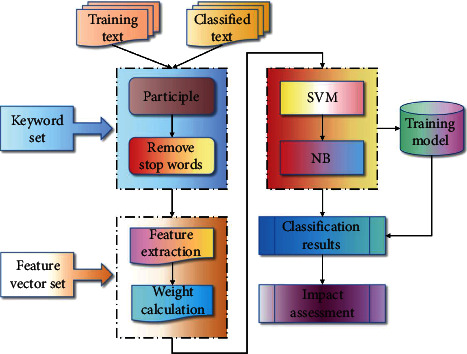
Text classification framework flowchart.

From this, it can be concluded that the process framework of the system prototype is shown in Figure 3.

In the traditional text classification technology, according to the research of related scholars, the SVM algorithm and NB algorithm have good classification effects and have been widely used in the field of text classification. Based on the statistical learning theory, SVM avoids the problem of infinite samples in traditional classification algorithms and has good generalization performance and obvious advantages in accuracy. At present, it has been successfully applied in the field of pattern recognition.

The excellent performance of SVM in nonlinear cases is largely due to its different inner product kernel functions. So far, there are three main types of kernel functions that are most used:

Linear kernel function:

(5) $$\begin{array}{c} K\left(x_{i},x_{j}\right)=x_{i}\cdot x_{j}. \end{array}$$

Polynomial kernel function:

(6) $$\begin{array}{c} K\left(x_{i},x_{j}\right)={\left[\left(x_{i}\cdot x_{j}\right)+1\right]}^{q}. \end{array}$$

The polynomial classifier of order *q* is obtained from the formula.

Radial basis kernel function:

(7) $$\begin{array}{c} K\left(x_{i},x_{j}\right)=\exp\left(-\gamma {\left\|x_{i}-x_{j}\right\|}^{2}\right). \end{array}$$

In this experiment, the radial basis function is chosen because it can also perform well for nonlinear mapping, and the complexity of the model depends on the number of parameters, which is small, so the model is not complicated, which is beneficial to text classification.

NB classifier is a classifier based on the Bayesian learning method and a classification method based on probability. Assuming that there are *m* classes *C*_1_, *C*_2_,…, *C*_*m*_, given the unknown text *d*, Bayesian classification will give the highest posterior probability of determining the class *C*_*i*_ for the text *d*, that is, maximizing *P*(*C*_*i*_*|d*). According to the Bayes theorem,

(8) $$\begin{array}{c} P\left(C_{i}|d\right)=\frac{P\left(d|C_{i}\right)\times P\left(C_{i}\right)}{P\left(d\right)}. \end{array}$$

Obviously, *P*(*d*) is a constant for all classes, so we just need to maximize *P*(*d|C*_*i*_) × *P*(*C*_*i*_). To avoid *P*(*C*_*i*_)=0, Laplace probability estimation is adopted as follows:

(9) $$\begin{array}{c} P\left(C_{i}\right)=\frac{1+\left|C_{i}\right|}{m+\left|D\right|}. \end{array}$$

|*C*_*i*_| is the number of texts contained in category *C*_*i*_, |*D*| is the total number of texts in the training set, and *P*(*d|C*_*i*_) is simply calculated by the probability of each attribute appearing in category *C*_*i*_.

Calculate the maximum posterior probability by the above formula:

(10) $$\begin{array}{c} y=\underset{c_{k}}{\arg\max}P\left(Y=c_{k}\right)\prod_{j=1}^{n}P\left(X^{\left(j\right)}=x^{\left(j\right)}|Y=c_{k}\right). \end{array}$$

When the calculated probability value of the category to which the document *x* belongs is the largest, it belongs to the category *c_k_*.

NB model has obvious advantages, its theoretical basis is classical mathematical theory, and its classification efficiency is stable. It is especially suitable for small-scale data and can solve the problem of multilabel classification. It can also be widely used in text classification, and its algorithm is simple and can be used when data is missing. There are a lot of hypothetical models in the field of ML, so the classification results may be biased due to the selection of prior models. At the same time, it is sensitive to the expression of input data.

## 4. Result Analysis

In this paper, NTCIR corpus is used for estimation and testing. NTCIR is a cross-language information retrieval conference dedicated to Asian languages, which provides the basic corpus of consulting retrieval and natural language processing, including a large number of Chinese news corpora. Select 500 articles from NTCIR Chinese corpus (all highly related or related to a certain topic) as the experimental corpus. In order to facilitate the evaluation of the results, these 500 corpora are manually labeled, and no more than 15 keywords are labeled for each document as a comparison standard by correcting and supplementing the corresponding topic keywords of each document.

The remaining 200 corpora are used as test corpora to test the performance of the keyword extraction method in this paper.

The following four groups of experiments were designed:

1. Only keywords being extracted.
2. Only keyword group extraction being carried out.
3. Appropriate combination of keywords and keyword phrases.
4. Similar to Experiment 3, except that word segmentation is not corrected.

The experimental results are shown in Table 1.

As can be seen from Table 1, the results of extracting only keywords or key phrases are not ideal. The combination of keywords and key phrases used in the text is more effective. And after the word segmentation result is optimized through the extraction process, the keyword extraction result is improved by nearly 2%, which shows that the merging process is quite meaningful.

**Figure 4:**
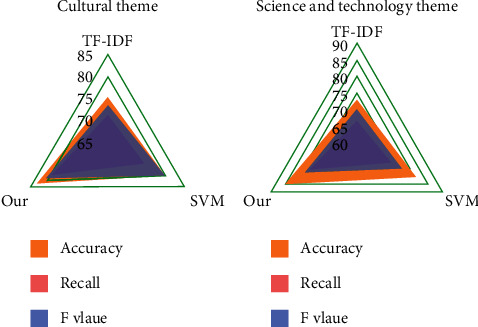
Comparison of topic sentence extraction effects under different methods.

In order to verify the effectiveness of this method, the TF-IDF method, SVM, and this method are used for comparative experiments. The performance of this method is verified by comparing the extraction results of the three methods. The experimental results are shown in Figure 4.

The experimental results show that the method in this paper has obviously improved on three evaluation indexes. The extraction effect of the TF-IDF method is the worst compared with the other two methods. The reason is that the nonevent sentences are not considered, which leads to a decrease in efficiency. Besides the characteristics of sentences themselves, the thematic relevance and semantic relevance between sentences and titles are also considered. Compared with the SVM method, the extraction effect of this method is still improved, which is mainly due to the comprehensive consideration of the correlation between events and the identification of the authenticity of event sentences.

This paper is a system based on candidate parallel resources filtering. This system also builds wrappers based on character surface features and then defines a sorting method based on length, bilingual dictionary, and translation model, which sorts all candidate resources, and the last one is noise data.  Table 2 shows the performance comparison results of English and American literature parallel resource acquisition systems.

As can be seen from Table 2, the quality of English and American literature corpus obtained by the method proposed in this section has been greatly improved, and the recall rate has also been slightly improved. According to statistics, the accuracy rate of parallel resources obtained by the system of [18] is only 71.66%, except for a few errors caused by improper text segmentation, and most other noises are introduced by high-quality templates themselves. The accuracy of this system is 82.24%. Compared with other systems, it is about 8%. Compared with the system of [18], the *F* value of the system of [20] has been improved to some extent.

Once the system identifies a wrapper as a nonquality type, it ignores its acquired parallel resources, but the ignored resources still contain parallel resources [18]. This system does not consider the quality of wrappers but takes all the resources acquired by wrappers as candidates, thus slightly improving the recall rate.

**Figure 5:**
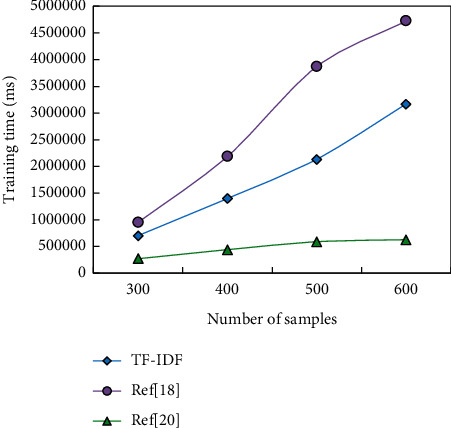
Time-consuming situation of training stage under different sample numbers.

For the second classification problem, a balanced number of samples is used for model training and text classification prediction; that is, the number of training samples and test samples is the same, and the number of samples in each category is the same. The TF-IDF text representation model of the corpus itself is a large and sparse matrix, which is linearly separable, so it is more suitable to use linear kernel function, and it does not need high-dimensional mapping. However, after the corpus is represented by reference [18] text and reference [20] text, the dimensions of the document model are greatly reduced, and the correctness of this inference is also verified by experiments.  Figure 5 shows the classification accuracy when the number of training samples and test samples is 300.

When matching, define the sequence length of the longest word in the dictionary for segmentation. On this basis, match it with the words in the dictionary. After the first word is cut out, repeat the remaining sequence according to the above method. However, due to the complexity of Chinese, the segmentation results obtained by the forward maximum matching algorithm are often not ideal, while the application of the reverse maximum matching algorithm will get a better segmentation result.

**Figure 6:**
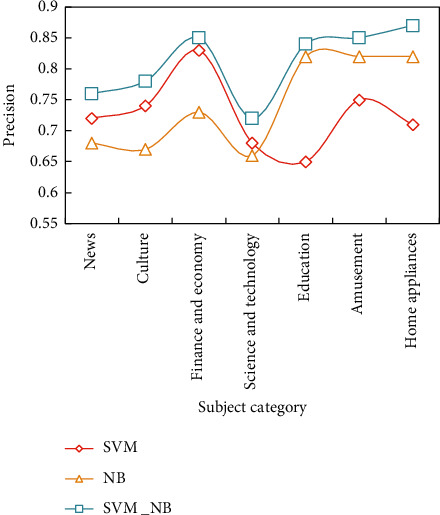
Precision of three classification methods.

**Figure 7:**
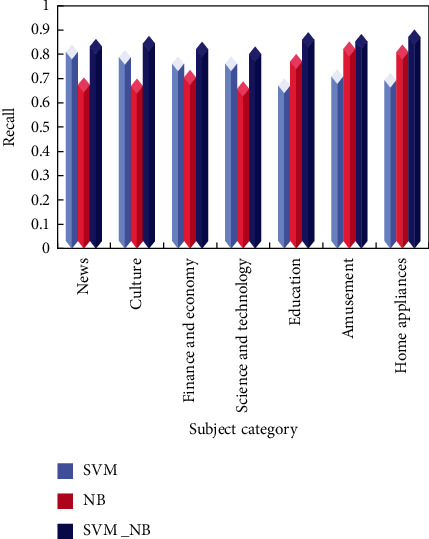
Recall rate of three classification methods.

**Figure 8:**
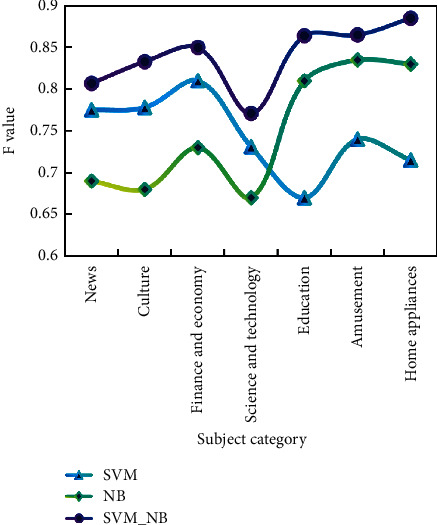
*F* values of three classification methods.

Test the classification effect of the combination classifier, that is, test SVM_NB, the combination classifier of SVM and NB, respectively. The classification details are shown in Figures 6–8.

On the whole, the recall rate of the three classification methods is slightly higher than the accuracy rate, and the SVM algorithm is better than the NB algorithm. The combination algorithm of SVM and NB is the best among the three, showing good accuracy and precision in both precision and recall.

At the same time, it is noted that when the SVM classifier is used to classify the small categories in the category of “Science and Technology,” the precision rate and recall rate are lower than those of the large categories. The classifier is also effective in classifying small classes.

It is concluded that the combined classifier combines the advantages of two classical classifiers and shows a very good classification effect when the text content is relatively independent. In terms of accuracy and recall, the classification effect of the combined algorithm proposed in this paper is the best among the three methods, and the best classification results of accuracy, recall, and *F* value are 0.87, 0.9, and 0.89, respectively.

## 5. Conclusion

In China, the application of corpus to teaching is still in its infancy. In this paper, the verification of bilingual mixed web pages is regarded as an effective classification problem, and the feature data based on length, translation degree of overlapping words, and word frequency are collected to train an effective classifier. In the process of extraction, we make full use of the characteristics of the web page itself, parts of speech, and other features, estimate the combination of key phrases and keywords through experiments, and achieve good keyword extraction results. A combined classifier of SVM and NB is constructed, and classification experiments are carried out on text corpora in different fields. The precision, recall, and *F* value of the proposed combined algorithm are 0.87, 0.9, and 0.89, respectively, and good classification results are achieved. There are still some areas to be improved in our English and American literature corpus; for example, the amount of information and retrieval methods need to be expanded and updated.

## Figures and Tables

**Table 1:** Experimental result.

Experimental project	Precision (%)	Recall %)	*F* value(%)
Experiment 1	24.13	28.66	26.87
Experiment 2	18.11	23.17	20.56
Experiment 3	34.32	44.05	38.29
Experiment 4	32.65	41.36	36.55

**Table 2:** Performance comparison results of parallel literature resource acquisition systems in Britain and America.

System name	Precision (%)	Recall (%)	*F* value (%)
Reference [18]	71.66	85.02	77.89
Reference [20]	73.63	85.11	78.96
This paper system	82.24	89.65	85.47

## Data Availability

The data set can be obtained from the author upon request.
